# Socioeconomic status and risk of lung cancer by histological subtype in the Nordic countries

**DOI:** 10.1002/cam4.4548

**Published:** 2022-02-15

**Authors:** Margherita Pizzato, Jan Ivar Martinsen, Sanna Heikkinen, Jerome Vignat, Elsebeth Lynge, Pär Sparén, Carlo La Vecchia, Eero Pukkala, Salvatore Vaccarella

**Affiliations:** ^1^ Department of Clinical Sciences and Community Health Università degli Studi di Milano Milan Italy; ^2^ Department of Research Cancer Registry of Norway Oslo Norway; ^3^ Finnish Cancer Registry Helsinki Finland; ^4^ International Agency for Research on Cancer Lyon France; ^5^ Nykøbing Falster Hospital University of Copenhagen Denmark; ^6^ Department of Medical Epidemiology and Biostatistics Karolinska Institutet Stockholm; ^7^ Finnish Cancer Registry Institute for Statistical and Epidemiological Cancer Research Helsinki Finland; ^8^ Faculty of Social Sciences, Tampere University Tampere Finland

**Keywords:** lung cancer, Nordic countries, socioeconomic status

## Abstract

**Background:**

While the excess in lung cancer risk among lower socioeconomic status individuals has been widely described, the magnitude of this association across lung cancer subtypes, as well as histotype‐related long‐term incidence trends, are inconclusively reported.

**Aims:**

We explored the variation in the incidence of the three main lung cancer histotypes (i.e. squamous cell carcinoma, small cell carcinoma and adenocarcinoma) by socioeconomic status (SES, i.e. upper and lower white collar, upper and lower blue collar, and farming/forestry/fishing) in the adult population of four Nordic countries (i.e. Sweden, Norway, Finland and Denmark).

**Materials & Methods:**

We have used data from the Nordic Occupational Cancer Study (NOCCA), computing age‐standardized incidence rates per 100,000 person‐years truncated at ages 50–69 years, by sex, histotype, country and SES, for the period 1971–2005. We estimated relative risks and the corresponding 95% confidence intervals through Poisson regression models, including terms for SES, age, sex and country, as indicated.

**Results:**

A clear socioeconomic gradient, with a progressive increase in lung cancer risk as SES level decreases, was observed in all subtypes and in both sexes. Favourable lung cancer incidence trends were seen among men for squamous cell and small cell carcinomas, although for adenocarcinomas rates were increasing everywhere except for Finland. Among women, upward temporal trends were seen in all SES groups and for all subtypes, although rates increased to a greater extent for low, compared to high, SES, especially in Denmark and Norway. Farmers showed comparatively lower risks compared to other SES categories.

**Discussion:**

This prospective cohort study shows that substantial socioeconomic inequalities in the incidence of the most important lung cancer histotypes exist in the Nordic Countries, and that these inequalities are on the rise, especially among women.

**Conclusion:**

Smoking habits are likely to largely explain the observed social gradient for lung cancer histotypes in both sexes.

## Graphical abstract

There are substantial socioeconomic status (SES) inequalities in lung cancer incidence in the Nordic countries with higher rates found in lower SES individuals. Over the period 1971‐2005 lung cancer rates declined in men and the decrease was stronger for squamous cell carcinoma. In women rates increased, more markedly in lower SES. Relative inequalities were greater in women than in men, and in both sexes were stronger for small cell and squamous cell carcinomas compared to adenocarcinoma.

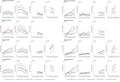

## 1 INTRODUCTION

Lung cancer encompasses a variety of histologic subtypes with distinct biological features and clinical behaviours. The most common types are squamous cell carcinoma, small cell carcinoma and adenocarcinoma.^1^, ^2^ In the Nordic countries, lung cancer incidence rate has declined among men since the mid‐1980s, mainly driven by the decrease in squamous cell and small cell histotypes.^3^ Among women, all three subtypes have steadily risen, especially adenocarcinoma, before plateauing recently.^4^ One of the reasons for the increase in incidence of histology‐specific types in earlier decades was a strong decrease of the proportion of lung cancer with undefined histology.

Differences in lung cancer risk have been observed in relationship to multiple socioeconomic status (SES) descriptors (e.g. educational level, occupational class and income), with higher lung cancer incidence rates usually reported among lower SES individuals.^5^, ^6^, ^7^ While the association between SES and lung cancer risk has been widely described, far less is known about its magnitude across lung cancer histotypes^8^, ^9^ and how it has evolved over time.

This historical prospective study with 35‐year follow‐up aims to describe SES variation in the incidence of three main lung cancer histotypes (i.e. squamous cell carcinoma, small cell carcinoma and adenocarcinoma) in the adult population of 50–69 years in four Nordic countries (i.e. Sweden, Norway, Finland and Denmark), using population‐based register data from the Nordic Occupational Cancer Study (NOCCA, http://astra.cancer.fi/NOCCA) database [Data availability statement].

## 2 MATERIALS AND METHODS

### 2.1 Study population and data collection

The NOCCA Study linked the census data on occupation from the Nordic countries with information on cancer diagnoses from the respective cancer registries for the period from 1961 to 2005.^10^ The unique personal identity code assigned to all residents in the Nordic countries was used to link census and cancer data.

The study cohort enrolled over 14 million persons, aged 30–64 who were alive and living in the four selected Nordic countries on January 1st in the year after any computerized census (i.e. Denmark [1970], Norway [1960, 1970 and 1980], Finland [1970, 1980 and 1990] and Sweden [1960, 1970, 1980 and 1990]).

Census questionnaires, centrally coded and computerized in the national statistical offices, included questions related to economic activity and occupation.^11^ The occupation codes, recorded in the first census that the person participated in, were converted to a common classification with 53 occupational categories, plus an additional category of economically inactive/undefined persons. These occupational categories were furthermore merged into five groups according to SES (i.e. upper white collar [UWC], lower white collar [LWC], upper blue collar [UBC], lower blue collar [LBC] and farming/forestry/fishing [farmers]).^12^ This social classification follows the original classification from the census in the 1970s.^13^ It includes four ordinary classes (i.e. UWC, LWC, UBC, and LBC), plus an additional one (i.e. farmers) with the aim of reflecting the type of education level achieved, the assumed social prestige and the income.^14^ As a special feature of the Nordic societies, farmers and related works are kept as a separate group. More details are reported in Table 1.

**TABLE 1:** Coding of occupational categories according to socioeconomic status level

Upper white collar	Technical workers, physicians, dentists, teachers, administrators
Lower white collar	Laboratory assistants, nurses, religious workers, artistic workers, journalists, clerical workers, sales agents, shop workers, transport workers, drivers, postal workers, public safety workers
Upper blue collar	Assistant nurses, other health workers, miners and quarry workers, seamen, textile workers, shoe and leather workers, smelting workers, mechanics, plumbers, welders, electrical workers, wood workers, painters, bricklayers, printers, chemical process workers, food workers, beverage workers, tobacco workers, glass makers, engine operators, cooks and stewards, waiters, chimney sweeps, hairdressers, launderers
Lower blue collar	Other construction workers, packers, domestic assistants, building caretakers
Farmers/forestry/fishing	Farmers, gardeners, fishermen, forestry workers

National population‐based cancer registration started in 1943 in Denmark, in 1953 in Finland and Norway, and in 1958 in Sweden. The cancer registries include information on cancer diagnosis, topography and morphology.^11^ We included over 58,000 lung cancer cases.

The individuals in the NOCCA Study were followed‐up from January 1 of the year following the first available census through the date of emigration, death or until December 31, 2003 (Norway and Denmark) or 2005 (Finland and Sweden).

The NOCCA Study was approved according to the rules of each participating country.^11^ Statistical analyses were performed using software R (version 4.0.5).

### 2.2 Statistical analysis

We computed age‐standardized incidence rates (ASRs) based on the World standard population per 100,000 person‐years at the truncated (TASRs) 50–69 age group (5‐year span) and corresponding 95% confidence intervals (CIs) by sex, histology, country and SES from 1971 until 2005 (5‐year observation periods). We applied the Tiwari method based on the beta distribution to calculate the 95% CIs for standardized rates.^15^ For Denmark, data on small cell carcinoma were not available, and data on squamous cell carcinoma and adenocarcinoma were available only for the period 1981–1995. For Sweden, data on all selected subtypes were available from 1986 to 2005. The analyses were restricted to ages 50–69 years as this was the only common age group available for all periods in all four study countries with the exception of Denmark for which data were available only for the period 1991–1995.

We estimated relative risks (RRs) and 95% CIs using Poisson regression models, pooling all countries together for the three most recent five‐year calendar periods that is 1991–2005, with the exception of Denmark for which data were available only for the period 1991–1995. The number of incident cases was modelled as the dependent variable, with the log of the person‐years at risk defined as an offset. Models included terms for SES (with UWC as reference group), age, sex and country.

## 3 RESULTS

**FIGURE 1:**
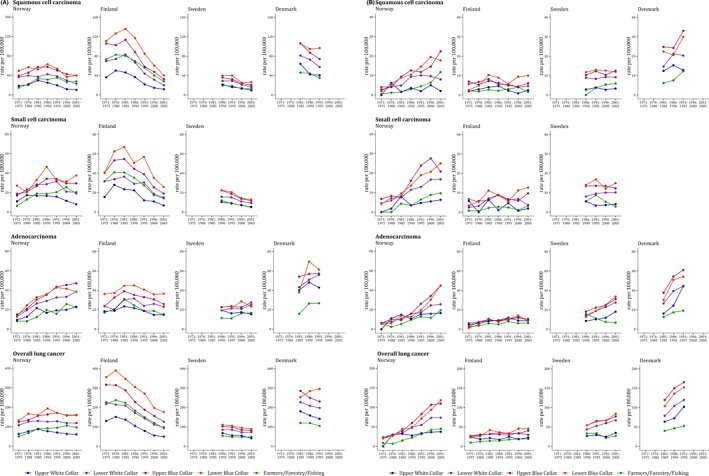
Age‐standardized incidence rates (world standard population) per 100,000 person‐years at the truncated 50–69 age group in men (A) and women (B) by lung cancer subtype, country and socioeconomic status, 1971–2005

Figure 1 displays the TASRs for the period 1971–2005 for men (a) and for women (b) aged 50–69 years by subtype, country and SES. The Supplementary Tables report TASRs and corresponding 95% CIs among men and women by lung cancer subtype and SES over the study period in Norway [Table S1], Finland [Table S2], Sweden [Table S3], Denmark [Table S4]. Over the whole observation period, a clear SES gradient was observed, consisting of a progressively higher lung cancer rates with lower SES, in all subtypes and in both sexes. The group of farmers, which cannot be located on the ordinal SES scale, showed overall intermediate to low rates. Among men [Figure 1A], squamous and small cell carcinoma rates decreased among all SES levels in all Nordic Countries, with Finland showing the highest rates and the strongest declines. After an initial increase, rates for squamous cell carcinoma in Finland declined from 136 cases per 100,000 in 1981–85 to 40/100,000 in 2001–2005 for LBC, and from 46/100,000 in 1981–1985 to 12/100,000 in 2001–2005 for UWC. Adenocarcinoma rates among men were generally increasing in all study countries, except for Finland where they increased only until the early 1970s, and then showed favourable trends in all SES two decades later, reaching in 2001–05 rates between 36/100,000 in LBC and 15/100,000 in Farmers. Among women [Figure 1B], lung cancer rates were generally increasing in all countries and SES groups, but with larger increases among lower SES groups. Increasing inequalities were particularly pronounced in Norway (squamous cell carcinoma: LBC, from 2/100,000 in 1971–1975 to 18/100,000 in 2001–2005; UBC, from 6/100,000 in 1976–1980 to 2/100,000 in 2001–2005; small‐cell carcinoma: LBC, from 5/100,000 in 1971–1975 to 25/100,000 in 2001–2005; UBC, from 2/100,000 in 1976–1980 to 6/100,000 in 2001–2005; adenocarcinoma: LBC, from 6/100,000 in 1971–1975 to 45/100,000 in 2001–2005; UBC, from 11/100,000 in 1976–1980 to 17/100,000 in 2001–2005) and Denmark (squamous cell carcinoma: LBC, from 22/100,000 in 1981–1985 to 30/100,000 in 1991–1995; UBC, from 12/100,000 in 1981–1985 to 13/100,000 in 1991–1995; adenocarcinoma: LBC, from 30/100,000 in 1981–1985 to 54/100,000 in 1991–1995; UBC, from 16/100,000 in 1981–1985 to 45/100,000 in 1991–1995) for all histotypes, and in Sweden for adenocarcinomas (LBC, from 13/100,000 in 1986–1990 to 34/100,000 in 2001–2005; UBC, from 9/100,000 in 1986–1990 to 18/100,000 in 2001–2005).

**FIGURE 2:**
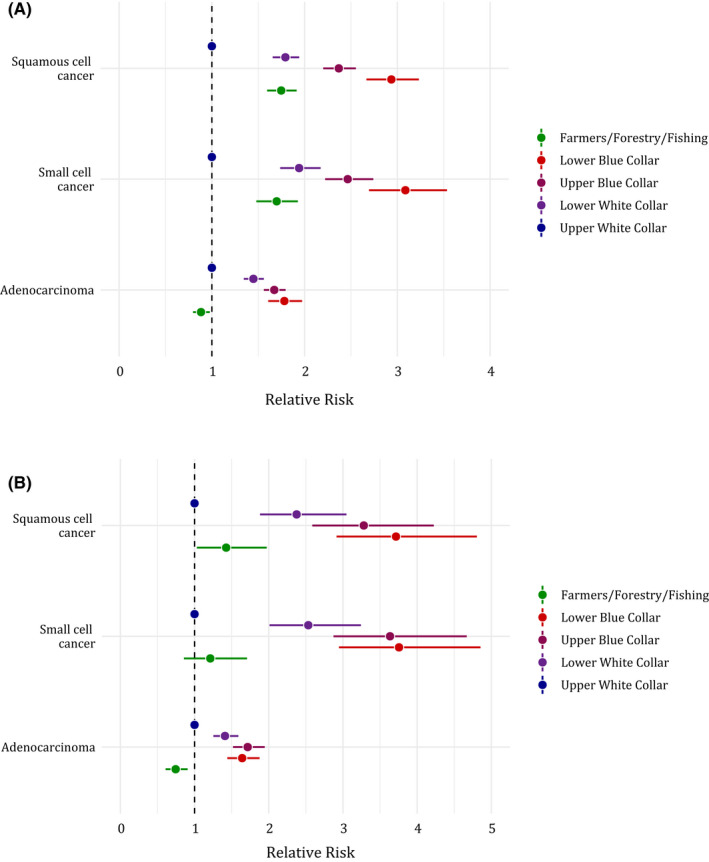
Relative risks (RRs) and corresponding 95% confidence intervals (CIs) of selected lung cancer histotypes in men (A) and in women (B) according to socioeconomic status, all four Nordic countries combined, 1991–2005 (except for Denmark 1991–1995)

Figure 2 displays the RRs and corresponding CIs of lung cancer incidence in 2001–2005, by histotype and SES group, with UWC as the reference group, for men (a) and women (b). There was a general increase in RR with decreasing SES level, for all subtypes and sexes. Regardless of sex, SES gradient appeared stronger in small cell (RRs for LBC were 3.09 [95% CI 2.70–3.54] in men and 3.76 [95% CI 2.95–4.85] in women) and squamous cell carcinomas (RRs for LBC were 2.94 [95% CI 2.67–3.23] in men and 3.72 [95% CI 2.91–4.81] in women) compared to adenocarcinoma (RRs for LBC were 1.78 [95% CI 1.61–1.97] in men and 1.64 [95% CI 1.44–1.88] in women). The farmers showed in both sexes an increased risk of small cell (RRs were 1.70 [95% CI 1.48–1.93] in men and 1.21 [95% CI 0.86–1.71] in women) and squamous cell carcinomas (RRs were 1.75 [95% CI 1.60–1.91] in men and 1.43 [95% CI 1.03–1.97] in women), but lower risk of adenocarcinoma (RRs were 0.88 [95% CI 0.80–0.98] in men and 0.75 [95% CI 0.61–0.91] in women) than any of the other SES categories.

## 4 DISCUSSION

This historical cohort study showed that decreasing levels of SES are associated with an excess in risk for all three selected lung cancer subtypes. Relative inequalities in both sexes were stronger for small cell and squamous cell carcinomas than for adenocarcinoma and the category of farmers felt among the groups with lowest risk. Gender disparities were also observed in temporal trends of lung cancer incidence: in brief, whereas mostly favourable trends were seen among men, especially for squamous cell and small cell carcinomas, rates among women were either stable or increased in all countries and for all histological subtypes, with more markedly increases in lower SES groups.

Several studies worldwide have addressed socioeconomic inequalities in lung cancer incidence with respect to individual SES indicators (e.g. level of education, income, occupation^16^), as well as to area‐based measures (e.g. neighbourhood deprivation^17^, ^18^, ^19^). These analyses described a consistent association between social position and incidence of lung cancer, with the lowest SES groups being those with the highest risk of developing lung cancer over the last several decades, even when major confounders were controlled.^5^, ^20^ In particular, when SES was assessed based on adult occupational class, an excess in risk was seen in both sexes related to decreasing occupational position.^21^, ^22^, ^23^

Despite well‐known etiological and clinical differences across lung cancer histotypes, only a few studies have dealt with social inequalities in histological terms and, to the best of our knowledge, none have explored histotype‐related long‐term trends in both sexes. A Canadian cohort study observed a negative gradient in lung cancer risk by occupational levels, with semi‐skilled/unskilled individuals being at the highest risk. This association was stronger for squamous cell and small cell carcinomas than for adenocarcinoma.^24^ Similarly, in an Australian cohort of women, the association between the area‐based index of education and occupation (IEO) and lung cancer histotypes, the incidence was smallest in adenocarcinoma.^25^ Even after adjustment for smoking habit and occupational exposures, lower occupational prestige in men was found more strongly related to squamous cell and small cell carcinomas, than to adenocarcinoma.^26^ An English study on 78,485 lung cancer cases, using an area‐based deprivation index as SES measure, showed a similar subtype‐related SES pattern in both sexes.^8^ Therefore, in line with our results, lower SES groups had a higher risk of being diagnosed with lung cancer^27^ and, regardless of SES descriptors, this negative social gradient was stronger for squamous cell and small cell carcinomas than for adenocarcinoma.

Multiple interrelated pathways may explain social inequalities in development of lung cancer by subtype.^28^, ^29^ The disproportional exposure to well‐known risk factors across social strata being one of the leading.^30^, ^31^ Tobacco smoking is the main risk factor for lung cancer, accounting for over 80% of incident lung cancer cases in the Nordic countries.^10^, ^32^ Despite some diversity in smoking exposure observed between Nordic regions, a common pattern of smoking across SES emerged in the last century. Smoking was initially adopted in higher social groups, before turning into a common habit equally widespread.^33^ From the middle of the last century, smoking prevalence has started to decline in all SES groups, albeit earlier and faster among individuals with higher SES.^34^, ^35^ As a result, cigarette smoking has become more common among lower SES groups,^36^ and hence, lung cancer has turned from a disease of higher SES to a disease of lower SES. Smoking patterns in women have temporally lagged behind those observed in men, for example higher SES Finnish women began smoking in the 1960s and started to quit a decade later, when low‐SES women only started to smoke.^37^ While cigarette smoking prevalence has remained steadily lower in women than in men, social discrepancy in tobacco consumption was more marked in females, at least for the more recent tobacco epidemic phases.^38^, ^39^ Persisting and widening SES differences seen among women are in line with tobacco epidemic characteristics in women, while increasing‐decreasing pattern observed in smoking‐related lung cancer subtypes reflected smoking consumption in males.^40^ The effects of smoking became visible in lung cancer risk after 20 years while the effects of quitting start more quickly, for example the rapid decrease in lung cancer incidence in Finnish men is due to massive stopping of smoking after 1970.^41^ Higher rates seen in both sexes over time in blue collar workers mirrored their attitude towards smoking, while low rates observed in farmers are likely attributable to the lower smoking prevalence found in rural areas. Relative inequalities were stronger for squamous cell and small cell carcinomas (i.e. lung cancer subtypes more related to older high tar cigarettes), and for these histotypes, disparities appeared more marked in women than in men. The widespread use of low tar cigarettes, which allow carcinogens to reach lung distal parts (i.e. where adenocarcinoma occurs^27^), along with new biopsy techniques improving access to lung periphery, explain the increase in adenocarcinoma diagnosis, as well as an increase of proportion of diagnoses with a histological confirmation.^42^ Historically, low tar cigarettes were consumed by upper social classes, due to their higher price; over most recent decades, they were more spread among women because of their lower tobacco tar content. This may explain the reduced SES inequalities seen for adenocarcinoma, especially in women.^43^, ^44^ Comparatively low rates of lung cancer observed among Swedish men are explained by the low prevalence of smoking; the widespread use of wet smokeless tobacco (i.e. snus) in this country partly substitutes tobacco smoking.^45^ Persistent disparities observed in Denmark may reflect tobacco policies conceived in that country, that appeared less stringent than those introduced in the other Nordic countries.^46^ Beyond individual tobacco consumption, passive exposure to environmental smoking in childhood was associated with households' lower education levels.^47^, ^48^, ^49^ This phenomenon has been found more prevalent among Danish parents, and, to some extent, attenuated in Finnish ones.^50^ Although smoking may explain a substantial part of the SES gradient in lung cancer incidence, tobacco exposure did not completely account for inequalities in lung cancer risk, since in several previous studies, after adjustment for smoking, a portion of the SES gradient still remains unexplained.^6^, ^51^ Although residual confounding from smoking may still exist, other mediators have been envisaged to explain the residual part of inequalities.^52^

Occupational exposures to workplace carcinogens (including asbestos, heavy metals and polycyclic aromatic hydrocarbons) account for a portion of the SES gradient in lung cancer, possibly explaining the higher rates observed in blue collar groups, especially men.^53^, ^54^ Outdoor air pollution, along with indoor exposure to carcinogens, may instead contribute to lung cancer incidence in lower SES individuals living in urban context.^55^, ^56^, ^57^ Other reasons for SES disparities are yet to be fully determined, but may consist of a combination of cultural factors (e.g. the attitude toward prevention or changing unhealthy behaviour), differences in lifestyle (e.g. selected dietary factors^58^), as well as disparities in medical assistance.^59^ Mechanisms related to healthcare, as well as an unequal access to broad and high‐quality medical services, should not be neglected when it comes to explaining SES inequalities in cancer incidence.^60^ However, considering that affordable and covering healthcare services are freely provided to all permanent residents in the Nordic countries, the potential contribution of a diagnostic bias is probably smaller than in other areas of the World.

Among the limitations of this study is the lack of data regarding individual smoking exposure, the use only of information on occupation recorded in the first available census, and the impossibility to explore variation on lifetime occupational history. No incidence data were available for periods more recent than 2005; in addition, data were unfortunately not available from all countries for all histological subtypes and periods. Difficulties in ascertaining and certifying lung cancer histotypes, especially in the past, may have affected the accuracy of incidence rates to a certain extent. Nonetheless, this large population‐based study includes among its main strengths the prospective design, the long and complete follow‐up, as well as the high quality and completeness of the data. In most studies on SES inequalities in health, the Nordic countries are often grouped together, while in this study we were able to explore trends by country. Moreover, SES indicators based on occupation encompass a wide spectrum of social information such as material resources, social status, and work‐related exposures. To the best of our knowledge, this is the largest cohort study using data from a long period of time to investigate SES inequalities by lung cancer histotypes.

In conclusion, this prospective cohort showed a substantial SES gradient in the incidence of lung cancer in the Nordic Countries, highlighting exacerbating socioeconomic and gender inequalities, and providing a quantification of these inequalities in terms of lung cancer histotypes.

## AUTHOR CONTRIBUTION

Margherita Pizzato, Salvatore Vaccarella, Sanna Heikkinen and Eero Pukkala contributed to the study conception and design. Material preparation and data acquisition were performed by all Nordic country authors. Data analysis was performed by Jan Ivar Martinsen, Jerome Vignat, Salvatore Vaccarella and Margherita Pizzato. Interpretation of data was performed by all authors. The first draft of the manuscript was written by Margherita Pizzato. All authors commented on previous versions of the manuscript and revising it critically. All authors read and approved the final manuscript.

## DISCLOSURE

All authors have declared no conflicts of interest.

## ETHICAL APPROVAL STATEMENT

The investigation did not involve any human contact, but only record linkage analysis of healthcare databases.

## Supporting information


Table S1

Table S2

Table S3

Table S4

## Data Availability

This historical prospective study was based on population‐based register data from the Nordic Occupational Cancer Study (NOCCA, http://astra.cancer.fi/NOCCA) database.
