# Supplementary material for: Socioeconomic status and risk of lung cancer by histological subtype in the Nordic countries
Source: Cancer Med. 2022 Feb 15;11(8):1850–9. doi: 10.1002/cam4.4548 (PMC9041078; doi:10.1002/cam4.4548)
Supplement: Supplementary file 1 — Table S1 [file CAM4-11-1850-s001.docx]

| **Table 1 Supplementary:** Age-standardized incidence rates (World standard population) [ASR] per 100,000 person-years at the truncated 50–69 age group and corresponding upper [UCI] and lower [LCI] 95% confidence intervals in Norway among men and women by lung cancer subtype and socioeconomic status, 1971-2005. | | | | | | | | | | | | | | | | | | | | | | | | | | | |
| --- | --- | --- | --- | --- | --- | --- | --- | --- | --- | --- | --- | --- | --- | --- | --- | --- | --- | --- | --- | --- | --- | --- | --- | --- | --- | --- | --- |
|  |  |  |  |  |  |  |  |  |  |  |  |  |  |  |  |  |  |  |  |  |  |  |  |  |  |  |  |
|  | **Squamous cell carcinoma** | | | | | |  | **Small cell carcinoma** | | | | | |  | **Adenocarcinoma** | | | | | |  | **Overall lung cancer** | | | | | |
|  | *Men* | | | *Women* | | |  | *Men* | | | *Women* | | |  | *Men* | | | *Women* | | |  | *Men* | | | *Women* | | |
|  | ASR | LCI | UCI | ASR | LCI | UCI |  | ASR | LCI | UCI | ASR | LCI | UCI |  | ASR | LCI | UCI | ASR | LCI | UCI |  | ASR | LCI | UCI | ASR | LCI | UCI |
| **Norway** |  |  |  |  |  |  |  |  |  |  |  |  |  |  |  |  |  |  |  |  |  |  |  |  |  |  |  |
| **Upper white collar** |  |  |  |  |  |  |  |  |  |  |  |  |  |  |  |  |  |  |  |  |  |  |  |  |  |  |  |
| 1971-1975 | 18.5 | 12.9 | 25.2 | 0 | 0 | 0 |  | 11.0 | 6.8 | 16.2 | 0 | 0 | 0 |  | 9.5 | 5.6 | 14.4 | 0 | 0 | 0 |  | 63.0 | 52.1 | 74.8 | 0 | 0 | 0 |
| 1976-1980 | 21.0 | 15.2 | 27.7 | 6.1 | 1.3 | 14.8 |  | 17.2 | 12.1 | 23.2 | 1.8 | 0 | 6.7 |  | 12.7 | 8.4 | 18.0 | 11.1 | 3.6 | 22.8 |  | 76.3 | 64.9 | 88.6 | 30.1 | 16.4 | 48.0 |
| 1981-1985 | 29.8 | 23.3 | 37.1 | 1.6 | 0 | 5.8 |  | 16.6 | 11.9 | 22.2 | 8.1 | 3.0 | 15.7 |  | 21.7 | 16.2 | 28.0 | 14.9 | 6.8 | 26.1 |  | 87.4 | 76.0 | 99.6 | 32.0 | 19.8 | 47.2 |
| 1986-1990 | 25.1 | 19.5 | 31.3 | 3.6 | 0.7 | 8.7 |  | 16.6 | 12.1 | 21.8 | 3.4 | 0.7 | 8.3 |  | 16.8 | 12.3 | 22.0 | 10.6 | 4.8 | 18.6 |  | 76.9 | 66.9 | 87.7 | 27.7 | 17.6 | 40.2 |
| 1991-1995 | 19.7 | 14.9 | 25.1 | 2.0 | 0.2 | 5.5 |  | 15.8 | 11.5 | 20.7 | 4.7 | 1.5 | 9.7 |  | 19.3 | 14.7 | 24.6 | 15.4 | 8.8 | 23.7 |  | 70.4 | 61.1 | 80.3 | 33.4 | 23.2 | 45.4 |
| 1996-2000 | 11.7 | 8.4 | 15.5 | 5.0 | 2.1 | 9.3 |  | 11.5 | 8.3 | 15.2 | 5.5 | 2.2 | 10.3 |  | 19.8 | 15.5 | 24.5 | 15.8 | 9.8 | 23.2 |  | 63.1 | 55.2 | 71.5 | 36.4 | 26.9 | 47.4 |
| 2001-2005 | 10.4 | 6.7 | 14.8 | 2.1 | 0.3 | 5.8 |  | 7.9 | 4.9 | 11.7 | 6.3 | 2.2 | 12.5 |  | 23.0 | 17.5 | 29.2 | 16.5 | 9.3 | 25.8 |  | 60.9 | 51.7 | 70.9 | 37.4 | 25.8 | 51.1 |
| **Lower white collar** |  |  |  |  |  |  |  |  |  |  |  |  |  |  |  |  |  |  |  |  |  |  |  |  |  |  |  |
| 1971-1975 | 36.6 | 30.9 | 42.9 | 4.0 | 2.1 | 6.5 |  | 16.9 | 13.1 | 21.2 | 4.3 | 2.3 | 6.8 |  | 12.9 | 9.6 | 16.7 | 6.5 | 4.0 | 9.5 |  | 107.8 | 97.7 | 118.4 | 23.1 | 18.1 | 28.7 |
| 1976-1980 | 39.6 | 33.9 | 45.8 | 4.3 | 2.5 | 6.6 |  | 23.1 | 18.8 | 27.8 | 7.1 | 4.7 | 9.9 |  | 19.4 | 15.5 | 23.8 | 7.8 | 5.3 | 10.8 |  | 126.3 | 116.0 | 137.1 | 30.0 | 24.8 | 35.6 |
| 1981-1985 | 37.5 | 32.1 | 43.2 | 4.8 | 3.2 | 6.9 |  | 28.2 | 23.6 | 33.3 | 7.6 | 5.4 | 10.1 |  | 26.3 | 21.8 | 31.1 | 10.8 | 8.2 | 13.7 |  | 129.5 | 119.3 | 140.1 | 33.8 | 29.0 | 38.9 |
| 1986-1990 | 42.5 | 36.8 | 48.6 | 9.4 | 7.1 | 11.9 |  | 28.6 | 24.0 | 33.7 | 11.4 | 8.9 | 14.3 |  | 28.8 | 24.1 | 34.0 | 15.8 | 12.8 | 19.2 |  | 125.8 | 115.9 | 136.2 | 49.3 | 43.8 | 55.0 |
| 1991-1995 | 38.2 | 32.7 | 44.1 | 10.1 | 7.8 | 12.6 |  | 31.4 | 26.4 | 36.7 | 12.9 | 10.3 | 15.9 |  | 32.5 | 27.5 | 37.9 | 17.2 | 14.2 | 20.6 |  | 127.8 | 117.7 | 138.4 | 55.0 | 49.4 | 60.9 |
| 1996-2000 | 29.8 | 25.1 | 35.0 | 9.7 | 7.5 | 12.2 |  | 20.9 | 17.0 | 25.2 | 16.7 | 13.8 | 19.8 |  | 33.1 | 28.1 | 38.5 | 24.5 | 20.9 | 28.3 |  | 119.0 | 109.3 | 129.1 | 73.3 | 67.0 | 79.8 |
| 2001-2005 | 22.8 | 17.6 | 28.6 | 8.0 | 5.4 | 11.0 |  | 20.3 | 15.4 | 25.8 | 16.8 | 13.0 | 21.1 |  | 38.5 | 31.6 | 45.9 | 25.6 | 20.9 | 30.8 |  | 119.1 | 106.8 | 131.9 | 73.5 | 65.3 | 82.1 |
| **Upper blue collar** |  |  |  |  |  |  |  |  |  |  |  |  |  |  |  |  |  |  |  |  |  |  |  |  |  |  |  |
| 1971-1975 | 39.4 | 35.0 | 44.2 | 2.8 | 1.1 | 5.3 |  | 18.7 | 15.7 | 22.0 | 6.8 | 3.9 | 10.4 |  | 15.0 | 12.3 | 18.0 | 6.1 | 3.4 | 9.6 |  | 124.5 | 116.5 | 132.8 | 20.5 | 15.2 | 26.5 |
| 1976-1980 | 45.6 | 41.0 | 50.5 | 4.0 | 2.0 | 6.8 |  | 20.7 | 17.6 | 24.0 | 8.3 | 5.1 | 12.1 |  | 20.9 | 17.8 | 24.2 | 10.3 | 6.7 | 14.8 |  | 134.9 | 126.9 | 143.2 | 29.6 | 23.4 | 36.6 |
| 1981-1985 | 57.1 | 52.0 | 62.5 | 9.4 | 6.3 | 13.0 |  | 26.7 | 23.3 | 30.4 | 7.8 | 5.1 | 11.0 |  | 30.3 | 26.6 | 34.2 | 9.3 | 6.2 | 13.0 |  | 155.7 | 147.1 | 164.5 | 39.0 | 32.5 | 46.1 |
| 1986-1990 | 57.3 | 52.2 | 62.7 | 12.6 | 8.9 | 17.0 |  | 34.2 | 30.2 | 38.3 | 16.1 | 12.0 | 20.9 |  | 35.2 | 31.1 | 39.5 | 15.9 | 11.9 | 20.5 |  | 163.2 | 154.4 | 172.3 | 59.7 | 51.4 | 68.5 |
| 1991-1995 | 50.2 | 45.3 | 55.3 | 11.8 | 8.2 | 16.0 |  | 33.9 | 29.9 | 38.2 | 23.8 | 18.7 | 29.6 |  | 43.5 | 38.9 | 48.3 | 26.1 | 20.6 | 32.3 |  | 171.4 | 162.2 | 180.8 | 82.9 | 72.9 | 93.5 |
| 1996-2000 | 42.7 | 38.2 | 47.5 | 14.6 | 10.6 | 19.1 |  | 29.4 | 25.7 | 33.4 | 27.6 | 21.9 | 33.8 |  | 45.4 | 40.7 | 50.3 | 34.1 | 27.8 | 41.1 |  | 157.7 | 149.0 | 166.8 | 106.6 | 95.2 | 118.6 |
| 2001-2005 | 39.6 | 34.0 | 45.5 | 22.4 | 15.9 | 30.0 |  | 29.4 | 24.6 | 34.7 | 20.8 | 14.7 | 28.0 |  | 47.0 | 40.8 | 53.7 | 44.6 | 35.1 | 55.3 |  | 159.3 | 147.7 | 171.2 | 109.6 | 94.4 | 125.9 |
| **Lower blue collar** |  |  |  |  |  |  |  |  |  |  |  |  |  |  |  |  |  |  |  |  |  |  |  |  |  |  |  |
| 1971-1975 | 49.4 | 37.5 | 62.9 | 1.9 | 0.5 | 4.2 |  | 27.1 | 18.4 | 37.5 | 4.6 | 2.2 | 7.8 |  | 13.2 | 7.4 | 20.8 | 5.6 | 2.9 | 9.3 |  | 131.9 | 111.8 | 153.6 | 20.5 | 14.9 | 27.0 |
| 1976-1980 | 56.7 | 43.6 | 71.4 | 5.5 | 3.0 | 8.8 |  | 20.2 | 12.9 | 29.2 | 5.9 | 3.1 | 9.6 |  | 24.5 | 16.1 | 34.7 | 6.1 | 3.3 | 9.7 |  | 166.3 | 143.0 | 191.2 | 26.5 | 20.3 | 33.5 |
| 1981-1985 | 52.2 | 39.4 | 66.9 | 8.9 | 6.0 | 12.4 |  | 32.9 | 22.9 | 44.7 | 9.6 | 6.4 | 13.4 |  | 32.7 | 22.7 | 44.6 | 13.7 | 9.9 | 18.1 |  | 159.9 | 136.5 | 185.1 | 44.5 | 37.4 | 52.2 |
| 1986-1990 | 62.9 | 47.9 | 79.8 | 10.4 | 7.1 | 14.4 |  | 46.4 | 33.8 | 60.9 | 13.6 | 9.8 | 18.1 |  | 35.8 | 24.7 | 48.9 | 12.7 | 9.0 | 17.1 |  | 193.7 | 166.8 | 222.6 | 46.9 | 39.5 | 54.9 |
| 1991-1995 | 53.9 | 40.1 | 69.8 | 14.5 | 10.5 | 19.1 |  | 33.1 | 22.4 | 45.8 | 18.9 | 14.1 | 24.5 |  | 42.7 | 30.3 | 57.2 | 19.6 | 14.6 | 25.4 |  | 170.7 | 145.0 | 198.5 | 71.2 | 61.6 | 81.6 |
| 1996-2000 | 36.7 | 25.6 | 49.9 | 19.5 | 14.5 | 25.1 |  | 31.3 | 21.1 | 43.5 | 20.7 | 15.5 | 26.7 |  | 41.5 | 29.6 | 55.4 | 30.3 | 23.9 | 37.4 |  | 159.5 | 135.2 | 185.8 | 93.8 | 82.3 | 106.0 |
| 2001-2005 | 39.9 | 25.4 | 57.5 | 17.7 | 11.3 | 25.6 |  | 37.5 | 23.3 | 55.0 | 25.0 | 17.4 | 33.9 |  | 38.4 | 23.9 | 56.2 | 44.8 | 33.3 | 58.1 |  | 161.2 | 130.1 | 195.6 | 118.2 | 99.5 | 138.4 |
| **Farmers/Forestry/Fishing** |  |  |  |  |  |  |  |  |  |  |  |  |  |  |  |  |  |  |  |  |  |  |  |  |  |  |  |
| 1971-1975 | 15.0 | 11.9 | 18.5 | 0.5 | 0 | 1.8 |  | 6.3 | 4.3 | 8.7 | 0 | 0 | 0 |  | 8.2 | 5.9 | 10.8 | 5.6 | 2.8 | 9.4 |  | 50.2 | 44.3 | 56.5 | 8.6 | 5.0 | 13.2 |
| 1976-1980 | 22.3 | 18.2 | 26.7 | 1.2 | 0.1 | 3.4 |  | 12.9 | 9.8 | 16.3 | 0 | 0 | 0 |  | 8.1 | 5.7 | 11.0 | 2.4 | 0.8 | 5.1 |  | 67.4 | 60.2 | 75.1 | 6.5 | 3.4 | 10.6 |
| 1981-1985 | 34.0 | 28.5 | 39.8 | 1.5 | 0.4 | 3.4 |  | 18.9 | 14.8 | 23.5 | 4.3 | 1.7 | 8.1 |  | 12.3 | 8.9 | 16.3 | 5.2 | 2.5 | 8.9 |  | 89.1 | 79.9 | 98.7 | 15.0 | 9.9 | 21.2 |
| 1986-1990 | 31.3 | 25.5 | 37.8 | 3.0 | 0.9 | 6.4 |  | 18.7 | 13.9 | 24.1 | 3.5 | 1.2 | 6.8 |  | 19.7 | 15.0 | 25.1 | 9.7 | 5.6 | 14.8 |  | 92.5 | 81.8 | 103.8 | 22.4 | 15.5 | 30.4 |
| 1991-1995 | 35.4 | 28.1 | 43.5 | 4.4 | 1.7 | 8.4 |  | 20.5 | 15.2 | 26.5 | 6.4 | 3.1 | 10.9 |  | 15.6 | 11.1 | 21.0 | 13.0 | 7.4 | 20.1 |  | 94.8 | 82.8 | 107.4 | 31.4 | 22.3 | 42.0 |
| 1996-2000 | 26.5 | 19.7 | 34.2 | 6.4 | 2.8 | 11.4 |  | 25.5 | 18.6 | 33.5 | 8.9 | 3.6 | 16.4 |  | 25.8 | 18.9 | 33.7 | 11.5 | 6.1 | 18.6 |  | 106.3 | 91.9 | 121.7 | 42.1 | 30.2 | 56.0 |
| 2001-2005 | 27.7 | 17.9 | 39.6 | 11.8 | 5.2 | 20.9 |  | 19.0 | 11.1 | 29.1 | 9.6 | 1.8 | 24.0 |  | 22.5 | 12.9 | 34.8 | 19.4 | 6.7 | 38.6 |  | 96.3 | 76.5 | 118.4 | 45.1 | 26.0 | 69.3 |
