# Supplementary material for: Socioeconomic status and risk of lung cancer by histological subtype in the Nordic countries
Source: Cancer Med. 2022 Feb 15;11(8):1850–9. doi: 10.1002/cam4.4548 (PMC9041078; doi:10.1002/cam4.4548)
Supplement: Supplementary file 2 — Table S2 [file CAM4-11-1850-s004.docx]

| **Table 2 Supplementary:** Age-standardized incidence rates (World standard population) [ASR] per 100,000 person-years at the truncated 50–69 age group and corresponding upper [UCI] and lower [LCI] 95% confidence intervals in Finland among men and women by lung cancer subtype and socioeconomic status, 1971-2005. | | | | | | | | | | | | | | | | | | | | | | | | | | | |
| --- | --- | --- | --- | --- | --- | --- | --- | --- | --- | --- | --- | --- | --- | --- | --- | --- | --- | --- | --- | --- | --- | --- | --- | --- | --- | --- | --- |
|  |  |  |  |  |  |  |  |  |  |  |  |  |  |  |  |  |  |  |  |  |  |  |  |  |  |  |  |
|  | **Squamous cell carcinoma** | | | | | |  | **Small cell carcinoma** | | | | | |  | **Adenocarcinoma** | | | | | |  | **Overall lung cancer** | | | | | |
|  | *Men* | | | *Women* | | |  | *Men* | | | *Women* | | |  | *Men* | | | *Women* | | |  | *Men* | | | *Women* | | |
|  | ASR | LCI | UCI | ASR | LCI | UCI |  | ASR | LCI | UCI | ASR | LCI | UCI |  | ASR | LCI | UCI | ASR | LCI | UCI |  | ASR | LCI | UCI | ASR | LCI | UCI |
| **Finland** |  |  |  |  |  |  |  |  |  |  |  |  |  |  |  |  |  |  |  |  |  |  |  |  |  |  |  |
| **Upper white collar** |  |  |  |  |  |  |  |  |  |  |  |  |  |  |  |  |  |  |  |  |  |  |  |  |  |  |  |
| 1971-1975 | 36.2 | 25.7 | 48.4 | 2.0 | 0.0 | 7.3 |  | 15.5 | 8.2 | 25.0 | 5.8 | 0.1 | 21.2 |  | 18.4 | 10.1 | 29.0 | 4.8 | 1.0 | 11.6 |  | 130.6 | 107.9 | 155.4 | 23.9 | 9.2 | 45.5 |
| 1976-1980 | 50.4 | 40.0 | 62.0 | 2.9 | 0.3 | 8.2 |  | 28.0 | 20.6 | 36.5 | 0.0 | 0.0 | 0.0 |  | 19.0 | 12.7 | 26.6 | 6.2 | 1.7 | 13.6 |  | 151.4 | 133.0 | 171.0 | 18.4 | 9.5 | 30.2 |
| 1981-1985 | 46.4 | 37.3 | 56.6 | 4.0 | 0.8 | 9.7 |  | 23.4 | 17.2 | 30.6 | 6.3 | 2.0 | 13.0 |  | 23.5 | 17.4 | 30.6 | 8.3 | 3.3 | 15.6 |  | 137.0 | 121.1 | 153.8 | 21.3 | 12.3 | 32.6 |
| 1986-1990 | 36.7 | 29.5 | 44.7 | 4.6 | 1.5 | 9.5 |  | 22.4 | 16.9 | 28.7 | 1.0 | 0.0 | 3.9 |  | 21.7 | 16.3 | 27.8 | 9.1 | 4.2 | 16.0 |  | 104.9 | 92.6 | 118.1 | 16.9 | 9.8 | 25.9 |
| 1991-1995 | 21.9 | 17.0 | 27.3 | 2.2 | 0.5 | 5.4 |  | 12.1 | 8.6 | 16.2 | 4.6 | 1.7 | 9.0 |  | 18.8 | 14.4 | 23.8 | 11.9 | 6.8 | 18.5 |  | 78.9 | 69.4 | 88.9 | 24.5 | 16.7 | 33.8 |
| 1996-2000 | 14.6 | 11.0 | 18.6 | 0.8 | 0.1 | 2.3 |  | 10.9 | 8.0 | 14.2 | 0.7 | 0.0 | 2.7 |  | 14.7 | 11.2 | 18.7 | 8.9 | 4.9 | 14.2 |  | 55.2 | 48.2 | 62.7 | 18.5 | 12.4 | 25.7 |
| 2001-2005 | 11.7 | 8.9 | 14.9 | 2.5 | 0.9 | 4.9 |  | 6.8 | 4.7 | 9.2 | 1.9 | 0.6 | 3.9 |  | 15.2 | 12.0 | 18.7 | 8.7 | 5.2 | 13.1 |  | 50.0 | 44.1 | 56.3 | 22.5 | 16.8 | 29.2 |
| **Lower white collar** |  |  |  |  |  |  |  |  |  |  |  |  |  |  |  |  |  |  |  |  |  |  |  |  |  |  |  |
| 1971-1975 | 69.8 | 58.0 | 82.6 | 5.6 | 2.5 | 9.9 |  | 31.6 | 23.6 | 40.7 | 3.2 | 0.8 | 7.4 |  | 23.6 | 17.3 | 30.9 | 6.2 | 3.3 | 10.0 |  | 226.8 | 205.2 | 249.3 | 26.8 | 19.3 | 35.6 |
| 1976-1980 | 74.1 | 64.9 | 83.9 | 6.8 | 4.3 | 9.7 |  | 33.8 | 27.8 | 40.4 | 5.7 | 3.5 | 8.5 |  | 19.9 | 15.3 | 25.2 | 7.3 | 4.9 | 10.3 |  | 214.9 | 199.0 | 231.3 | 30.4 | 25.0 | 36.4 |
| 1981-1985 | 83.3 | 74.3 | 92.7 | 8.2 | 5.8 | 11.0 |  | 36.5 | 30.7 | 42.9 | 6.1 | 4.1 | 8.6 |  | 30.3 | 25.0 | 36.1 | 9.0 | 6.5 | 11.8 |  | 209.5 | 195.1 | 224.4 | 31.7 | 26.8 | 37.0 |
| 1986-1990 | 66.9 | 59.5 | 74.7 | 5.3 | 3.6 | 7.4 |  | 29.0 | 24.3 | 34.2 | 8.9 | 6.6 | 11.4 |  | 31.5 | 26.6 | 36.9 | 9.3 | 7.0 | 11.9 |  | 172.2 | 160.3 | 184.6 | 30.7 | 26.3 | 35.4 |
| 1991-1995 | 43.9 | 38.4 | 49.7 | 5.6 | 4.0 | 7.5 |  | 30.5 | 26.0 | 35.4 | 6.2 | 4.5 | 8.2 |  | 23.9 | 20.0 | 28.2 | 9.4 | 7.2 | 11.8 |  | 143.7 | 133.7 | 154.0 | 29.7 | 25.8 | 34.0 |
| 1996-2000 | 31.3 | 27.0 | 35.9 | 4.0 | 2.7 | 5.4 |  | 18.8 | 15.5 | 22.4 | 6.7 | 5.0 | 8.6 |  | 26.1 | 22.3 | 30.3 | 12.7 | 10.4 | 15.2 |  | 111.4 | 103.2 | 120.0 | 33.9 | 30.1 | 38.0 |
| 2001-2005 | 20.0 | 16.8 | 23.5 | 4.6 | 3.4 | 6.1 |  | 15.0 | 12.3 | 18.0 | 3.7 | 2.6 | 4.9 |  | 23.1 | 19.7 | 26.9 | 8.9 | 7.2 | 10.7 |  | 97.3 | 90.0 | 104.8 | 29.9 | 26.7 | 33.3 |
| **Upper blue collar** |  |  |  |  |  |  |  |  |  |  |  |  |  |  |  |  |  |  |  |  |  |  |  |  |  |  |  |
| 1971-1975 | 106.0 | 94.8 | 117.8 | 6.9 | 3.0 | 12.3 |  | 40.5 | 34.1 | 47.4 | 2.4 | 1.0 | 4.5 |  | 23.6 | 18.7 | 29.2 | 2.5 | 0.6 | 5.7 |  | 317.0 | 297.2 | 337.5 | 25.1 | 17.8 | 33.7 |
| 1976-1980 | 102.7 | 93.8 | 112.0 | 5.3 | 3.1 | 8.1 |  | 53.3 | 47.0 | 60.0 | 3.2 | 1.6 | 5.4 |  | 32.6 | 27.7 | 37.8 | 7.5 | 4.9 | 10.6 |  | 314.6 | 298.9 | 330.8 | 29.1 | 23.6 | 35.0 |
| 1981-1985 | 113.7 | 104.8 | 122.9 | 7.2 | 4.9 | 10.0 |  | 54.6 | 48.6 | 61.0 | 7.1 | 4.8 | 9.8 |  | 39.1 | 34.1 | 44.5 | 10.1 | 7.3 | 13.3 |  | 288.7 | 274.5 | 303.2 | 32.0 | 26.8 | 37.6 |
| 1986-1990 | 88.5 | 81.3 | 95.9 | 6.3 | 4.3 | 8.7 |  | 44.5 | 39.5 | 49.8 | 8.9 | 6.4 | 11.7 |  | 35.3 | 30.9 | 40.1 | 8.1 | 5.8 | 10.9 |  | 228.3 | 216.7 | 240.2 | 34.3 | 29.3 | 39.7 |
| 1991-1995 | 58.1 | 52.7 | 63.7 | 4.9 | 3.2 | 6.9 |  | 39.0 | 34.6 | 43.7 | 6.6 | 4.6 | 9.0 |  | 32.9 | 28.9 | 37.2 | 11.2 | 8.4 | 14.4 |  | 188.2 | 178.3 | 198.3 | 32.9 | 28.1 | 38.1 |
| 1996-2000 | 47.5 | 42.9 | 52.4 | 4.4 | 2.8 | 6.4 |  | 25.5 | 22.1 | 29.0 | 5.9 | 4.0 | 8.1 |  | 30.7 | 27.0 | 34.6 | 11.8 | 9.0 | 14.9 |  | 156.5 | 148.0 | 165.2 | 33.0 | 28.3 | 38.1 |
| 2001-2005 | 32.4 | 28.8 | 36.2 | 6.0 | 4.1 | 8.2 |  | 19.0 | 16.3 | 22.0 | 9.7 | 7.3 | 12.4 |  | 25.9 | 22.7 | 29.3 | 10.4 | 7.9 | 13.2 |  | 126.1 | 118.9 | 133.6 | 40.5 | 35.4 | 45.9 |
| **Lower blue collar** |  |  |  |  |  |  |  |  |  |  |  |  |  |  |  |  |  |  |  |  |  |  |  |  |  |  |  |
| 1971-1975 | 111.3 | 92.1 | 132.3 | 2.5 | 0.6 | 6.0 |  | 40.3 | 29.0 | 53.5 | 6.7 | 2.9 | 12.2 |  | 36.2 | 24.8 | 49.6 | 1.3 | 0.3 | 3.1 |  | 356.0 | 320.3 | 393.5 | 22.8 | 15.5 | 31.5 |
| 1976-1980 | 126.8 | 110.2 | 144.6 | 4.8 | 2.5 | 7.9 |  | 62.5 | 51.1 | 75.1 | 5.3 | 2.8 | 8.6 |  | 37.3 | 28.6 | 47.1 | 5.0 | 2.6 | 8.2 |  | 390.8 | 361.2 | 421.5 | 26.3 | 20.2 | 33.1 |
| 1981-1985 | 136.1 | 119.4 | 153.9 | 10.4 | 6.7 | 14.7 |  | 67.1 | 55.5 | 79.7 | 11.1 | 7.4 | 15.5 |  | 44.7 | 35.4 | 55.0 | 10.8 | 7.2 | 15.2 |  | 346.9 | 319.9 | 375.0 | 41.8 | 34.2 | 50.1 |
| 1986-1990 | 117.1 | 102.0 | 133.2 | 8.9 | 5.8 | 12.8 |  | 50.7 | 41.0 | 61.5 | 8.6 | 5.3 | 12.8 |  | 45.1 | 35.9 | 55.3 | 7.6 | 4.6 | 11.4 |  | 305.5 | 280.8 | 331.2 | 36.0 | 28.8 | 43.9 |
| 1991-1995 | 85.0 | 72.3 | 98.7 | 5.7 | 3.2 | 9.1 |  | 56.8 | 46.5 | 68.2 | 5.5 | 3.1 | 8.7 |  | 40.7 | 32.1 | 50.4 | 10.7 | 6.9 | 15.3 |  | 271.4 | 248.2 | 295.5 | 33.6 | 26.7 | 41.3 |
| 1996-2000 | 60.7 | 49.8 | 72.7 | 9.4 | 5.9 | 13.8 |  | 35.2 | 27.1 | 44.4 | 11.2 | 7.4 | 15.7 |  | 35.9 | 27.6 | 45.3 | 14.2 | 9.9 | 19.2 |  | 197.6 | 177.6 | 218.8 | 45.8 | 37.7 | 54.6 |
| 2001-2005 | 40.3 | 31.5 | 50.1 | 10.0 | 6.6 | 14.0 |  | 25.8 | 18.7 | 34.0 | 12.6 | 8.8 | 17.1 |  | 36.5 | 28.1 | 45.8 | 8.5 | 5.5 | 12.3 |  | 176.3 | 157.2 | 196.5 | 45.5 | 37.9 | 53.7 |
| **Farmers/Forestry/Fishing** |  |  |  |  |  |  |  |  |  |  |  |  |  |  |  |  |  |  |  |  |  |  |  |  |  |  |  |
| 1971-1975 | 73.6 | 65.7 | 81.9 | 2.1 | 0.7 | 4.3 |  | 31.8 | 26.7 | 37.4 | 0.8 | 0.2 | 1.9 |  | 17.1 | 13.5 | 21.2 | 2.4 | 0.9 | 4.6 |  | 217.1 | 203.3 | 231.3 | 9.8 | 6.3 | 14.1 |
| 1976-1980 | 83.0 | 75.6 | 90.8 | 1.8 | 0.7 | 3.4 |  | 41.0 | 35.8 | 46.6 | 0.5 | 0.1 | 1.5 |  | 20.4 | 16.8 | 24.4 | 3.6 | 2.0 | 5.6 |  | 237.9 | 225.2 | 251.0 | 12.1 | 8.9 | 15.7 |
| 1981-1985 | 80.7 | 73.3 | 88.5 | 2.8 | 1.4 | 4.6 |  | 40.8 | 35.5 | 46.4 | 2.1 | 0.8 | 4.0 |  | 31.0 | 26.5 | 35.9 | 6.1 | 3.8 | 8.8 |  | 223.8 | 211.4 | 236.7 | 14.0 | 10.4 | 18.1 |
| 1986-1990 | 69.5 | 62.6 | 76.8 | 2.2 | 1.1 | 3.8 |  | 35.3 | 30.3 | 40.7 | 2.8 | 1.3 | 4.8 |  | 24.6 | 20.6 | 29.0 | 5.0 | 2.8 | 7.8 |  | 183.5 | 172.1 | 195.3 | 14.2 | 10.5 | 18.4 |
| 1991-1995 | 56.4 | 50.0 | 63.2 | 3.0 | 1.4 | 5.3 |  | 27.6 | 23.0 | 32.5 | 2.4 | 1.1 | 4.3 |  | 19.0 | 15.3 | 23.2 | 8.0 | 5.0 | 11.7 |  | 149.2 | 138.6 | 160.2 | 17.4 | 12.9 | 22.6 |
| 1996-2000 | 40.0 | 34.1 | 46.3 | 4.0 | 1.8 | 7.0 |  | 17.4 | 13.6 | 21.7 | 1.0 | 0.3 | 2.2 |  | 18.8 | 14.7 | 23.3 | 6.2 | 3.4 | 9.7 |  | 120.2 | 109.8 | 131.0 | 18.8 | 13.7 | 24.8 |
| 2001-2005 | 27.7 | 22.5 | 33.4 | 1.6 | 0.4 | 3.6 |  | 14.1 | 10.5 | 18.2 | 3.2 | 1.3 | 6.1 |  | 14.6 | 10.9 | 18.9 | 6.4 | 3.4 | 10.2 |  | 93.9 | 84.2 | 104.2 | 19.4 | 14.0 | 25.8 |
|  |  |  |  |  |  |  |  |  |  |  |  |  |  |  |  |  |  |  |  |  |  |  |  |  |  |  |  |
