# Supplementary material for: Socioeconomic status and risk of lung cancer by histological subtype in the Nordic countries
Source: Cancer Med. 2022 Feb 15;11(8):1850–9. doi: 10.1002/cam4.4548 (PMC9041078; doi:10.1002/cam4.4548)
Supplement: Supplementary file 3 — Table S3 [file CAM4-11-1850-s002.docx]

| **Table 3 Supplementary:** Age-standardized incidence rates (World standard population) [ASR] per 100,000 person-years at the truncated 50–69 age group and corresponding upper [UCI] and lower [LCI] 95% confidence intervals in Sweden among men and women by lung cancer subtype and socioeconomic status, 1986-2005. | | | | | | | | | | | | | | | | | | | | | | | | | | | |
| --- | --- | --- | --- | --- | --- | --- | --- | --- | --- | --- | --- | --- | --- | --- | --- | --- | --- | --- | --- | --- | --- | --- | --- | --- | --- | --- | --- |
|  |  |  |  |  |  |  |  |  |  |  |  |  |  |  |  |  |  |  |  |  |  |  |  |  |  |  |  |
|  | **Squamous cell carcinoma** | | | | | |  | **Small cell carcinoma** | | | | | |  | **Adenocarcinoma** | | | | | |  | **Overall lung cancer** | | | | | |
|  | *Men* | | | *Women* | | |  | *Men* | | | *Women* | | |  | *Men* | | | *Women* | | |  | *Men* | | | *Women* | | |
|  | ASR | LCI | UCI | ASR | LCI | UCI |  | ASR | LCI | UCI | ASR | LCI | UCI |  | ASR | LCI | UCI | ASR | LCI | UCI |  | ASR | LCI | UCI | ASR | LCI | UCI |
| **Sweden** |  |  |  |  |  |  |  |  |  |  |  |  |  |  |  |  |  |  |  |  |  |  |  |  |  |  |  |
| **Upper white collar** |  |  |  |  |  |  |  |  |  |  |  |  |  |  |  |  |  |  |  |  |  |  |  |  |  |  |  |
| 1986-1990 | 21.4 | 18.3 | 24.8 | 2.8 | 0.8 | 6.2 |  | 10.3 | 8.1 | 12.7 | 5.6 | 2.5 | 9.8 |  | 19.5 | 16.4 | 22.8 | 8.5 | 4.7 | 13.5 |  | 67.7 | 62.0 | 73.7 | 26.6 | 19.3 | 35.2 |
| 1991-1995 | 16.2 | 13.5 | 19.1 | 3.8 | 1.8 | 6.6 |  | 9.1 | 7.1 | 11.3 | 3.3 | 1.5 | 6.0 |  | 16.3 | 13.6 | 19.2 | 10.2 | 6.6 | 14.5 |  | 55.2 | 50.1 | 60.5 | 29.2 | 22.7 | 36.4 |
| 1996-2000 | 13.9 | 11.6 | 16.5 | 2.7 | 1.4 | 4.5 |  | 7.1 | 5.4 | 8.9 | 3.6 | 1.9 | 5.9 |  | 17.5 | 14.9 | 20.4 | 11.8 | 8.6 | 15.6 |  | 54.5 | 49.8 | 59.5 | 24.8 | 19.9 | 30.2 |
| 2001-2005 | 9.4 | 7.6 | 11.3 | 3.3 | 1.8 | 5.3 |  | 5.1 | 3.8 | 6.6 | 4.2 | 2.5 | 6.2 |  | 15.4 | 13.1 | 17.8 | 18.1 | 14.4 | 22.2 |  | 41.4 | 37.6 | 45.4 | 34.7 | 29.5 | 40.3 |
| **Lower white collar** |  |  |  |  |  |  |  |  |  |  |  |  |  |  |  |  |  |  |  |  |  |  |  |  |  |  |  |
| 1986-1990 | 28.8 | 25.8 | 32.1 | 8.9 | 7.0 | 10.9 |  | 15.8 | 13.5 | 18.2 | 8.1 | 6.3 | 10.1 |  | 19.4 | 16.9 | 22.1 | 14.4 | 11.9 | 17.0 |  | 85.1 | 79.7 | 90.6 | 43.4 | 39.1 | 47.9 |
| 1991-1995 | 29.1 | 26.0 | 32.3 | 8.3 | 6.6 | 10.2 |  | 15.1 | 12.9 | 17.5 | 9.5 | 7.7 | 11.6 |  | 21.5 | 18.8 | 24.3 | 18.8 | 16.2 | 21.5 |  | 85.0 | 79.6 | 90.5 | 52.8 | 48.4 | 57.4 |
| 1996-2000 | 21.3 | 18.7 | 24.1 | 9.3 | 7.6 | 11.1 |  | 10.9 | 9.1 | 12.9 | 10.1 | 8.4 | 12.0 |  | 21.0 | 18.4 | 23.7 | 22.9 | 20.3 | 25.6 |  | 71.4 | 66.6 | 76.4 | 59.8 | 55.5 | 64.1 |
| 2001-2005 | 16.2 | 14.0 | 18.5 | 9.2 | 7.7 | 10.8 |  | 9.2 | 7.6 | 11.0 | 10.2 | 8.6 | 11.9 |  | 25.1 | 22.4 | 27.9 | 27.6 | 25.1 | 30.3 |  | 72.9 | 68.3 | 77.6 | 67.0 | 62.9 | 71.1 |
| **Upper blue collar** |  |  |  |  |  |  |  |  |  |  |  |  |  |  |  |  |  |  |  |  |  |  |  |  |  |  |  |
| 1986-1990 | 35.8 | 32.9 | 38.8 | 10.3 | 7.7 | 13.2 |  | 22.3 | 20.0 | 24.8 | 13.5 | 10.5 | 16.7 |  | 22.6 | 20.3 | 25.1 | 18.2 | 14.8 | 22.0 |  | 102.6 | 97.6 | 107.7 | 54.2 | 48.1 | 60.7 |
| 1991-1995 | 32.4 | 29.6 | 35.3 | 12.3 | 9.5 | 15.4 |  | 18.7 | 16.6 | 21.0 | 13.5 | 10.7 | 16.7 |  | 23.7 | 21.3 | 26.3 | 22.2 | 18.5 | 26.3 |  | 98.9 | 94.0 | 104.0 | 65.5 | 58.9 | 72.4 |
| 1996-2000 | 23.7 | 21.3 | 26.2 | 10.4 | 8.1 | 13.1 |  | 13.7 | 11.9 | 15.6 | 13.5 | 10.8 | 16.5 |  | 22.6 | 20.3 | 25.0 | 24.7 | 21.0 | 28.7 |  | 83.5 | 79.0 | 88.2 | 66.9 | 60.7 | 73.4 |
| 2001-2005 | 20.5 | 18.3 | 22.7 | 12.5 | 10.1 | 15.2 |  | 11.7 | 10.1 | 13.4 | 12.3 | 9.9 | 14.9 |  | 27.5 | 25.0 | 30.1 | 31.1 | 27.3 | 35.2 |  | 82.7 | 78.4 | 87.2 | 77.2 | 71.1 | 83.6 |
| **Lower blue collar** |  |  |  |  |  |  |  |  |  |  |  |  |  |  |  |  |  |  |  |  |  |  |  |  |  |  |  |
| 1986-1990 | 39.9 | 32.4 | 48.0 | 11.8 | 7.7 | 16.6 |  | 22.5 | 17.0 | 28.8 | 14.0 | 9.6 | 19.3 |  | 19.3 | 14.1 | 25.4 | 12.8 | 8.7 | 17.6 |  | 110.5 | 97.7 | 124.2 | 54.1 | 45.1 | 63.9 |
| 1991-1995 | 40.0 | 32.1 | 48.6 | 12.9 | 9.0 | 17.5 |  | 20.5 | 15.1 | 26.8 | 16.9 | 12.2 | 22.2 |  | 21.2 | 15.7 | 27.7 | 18.8 | 14.1 | 24.1 |  | 105.7 | 92.7 | 119.6 | 64.1 | 55.0 | 74.0 |
| 1996-2000 | 25.2 | 19.1 | 32.1 | 12.6 | 9.1 | 16.7 |  | 14.4 | 9.9 | 19.7 | 12.1 | 8.8 | 16.0 |  | 28.6 | 22.2 | 35.9 | 25.1 | 20.2 | 30.5 |  | 95.2 | 83.0 | 108.2 | 66.7 | 58.6 | 75.4 |
| 2001-2005 | 26.4 | 20.4 | 33.2 | 11.9 | 9.0 | 15.2 |  | 12.8 | 8.8 | 17.5 | 14.9 | 11.6 | 18.7 |  | 23.3 | 17.7 | 29.7 | 33.7 | 28.7 | 39.0 |  | 88.5 | 77.2 | 100.6 | 84.0 | 76.0 | 92.5 |
| **Farmers/Forestry/Fishing** |  |  |  |  |  |  |  |  |  |  |  |  |  |  |  |  |  |  |  |  |  |  |  |  |  |  |  |
| 1986-1990 | 19.3 | 15.7 | 23.3 | 0 | 0 | 0 |  | 11.9 | 8.9 | 15.4 | 5.5 | 1.5 | 12.2 |  | 11.4 | 8.4 | 14.9 | 15.9 | 7.8 | 26.9 |  | 53.6 | 47.2 | 60.4 | 33.2 | 20.8 | 48.4 |
| 1991-1995 | 18.5 | 14.5 | 22.9 | 3.7 | 0.7 | 8.9 |  | 8.8 | 6.1 | 11.9 | 8.8 | 3.5 | 16.4 |  | 11.1 | 8.1 | 14.6 | 11.2 | 5.1 | 19.6 |  | 48.4 | 41.7 | 55.5 | 33.7 | 22.2 | 47.6 |
| 1996-2000 | 12.7 | 9.3 | 16.7 | 5.4 | 1.7 | 11.0 |  | 7.2 | 4.5 | 10.5 | 5.4 | 1.7 | 11.0 |  | 16.2 | 11.9 | 21.3 | 7.6 | 3.0 | 14.2 |  | 47.3 | 39.8 | 55.3 | 21.8 | 13.3 | 32.4 |
| 2001-2005 | 12.8 | 8.8 | 17.5 | 5.9 | 2.2 | 11.6 |  | 5.3 | 3.0 | 8.4 | 2.9 | 0.6 | 7.0 |  | 16.7 | 12.2 | 22.1 | 6.8 | 2.7 | 12.7 |  | 48.2 | 40.1 | 57.0 | 26.7 | 17.3 | 38.2 |
|  |  |  |  |  |  |  |  |  |  |  |  |  |  |  |  |  |  |  |  |  |  |  |  |  |  |  |  |
