# Supplementary material for: Socioeconomic status and risk of lung cancer by histological subtype in the Nordic countries
Source: Cancer Med. 2022 Feb 15;11(8):1850–9. doi: 10.1002/cam4.4548 (PMC9041078; doi:10.1002/cam4.4548)
Supplement: Supplementary file 4 — Table S4 [file CAM4-11-1850-s003.docx]

| **Table 4 Supplementary:** Age-standardized incidence rates (World standard population) [ASR] per 100,000 person-years at the truncated 50–69 age group and corresponding upper [UCI] and lower [LCI] 95% confidence intervals in Denmark among men and women by lung cancer subtype and socioeconomic status, 1981-1995. | | | | | | | | | | | | | | | | | | | | | | | | | | | |
| --- | --- | --- | --- | --- | --- | --- | --- | --- | --- | --- | --- | --- | --- | --- | --- | --- | --- | --- | --- | --- | --- | --- | --- | --- | --- | --- | --- |
|  |  |  |  |  |  |  |  |  |  |  |  |  |  |  |  |  |  |  |  |  |  |  |  |  |  |  |  |
|  | **Squamous cell carcinoma** | | | | | |  | **Small cell carcinoma** | | | | | |  | **Adenocarcinoma** | | | | | |  | **Overall lung cancer** | | | | | |
|  | *Men* | | | *Women* | | |  | *Men* | | | *Women* | | |  | *Men* | | | *Women* | | |  | *Men* | | | *Women* | | |
|  | ASR | LCI | UCI | ASR | LCI | UCI |  | ASR | LCI | UCI | ASR | LCI | UCI |  | ASR | LCI | UCI | ASR | LCI | UCI |  | ASR | LCI | UCI | ASR | LCI | UCI |
| **Denmark** |  |  |  |  |  |  |  |  |  |  |  |  |  |  |  |  |  |  |  |  |  |  |  |  |  |  |  |
| **Upper white collar** |  |  |  |  |  |  |  |  |  |  |  |  |  |  |  |  |  |  |  |  |  |  |  |  |  |  |  |
| 1981-1985 | 64.0 | 56.7 | 71.9 | 12.4 | 6.4 | 20.4 |  | - | - | - | - | - | - |  | 40.1 | 34.4 | 46.4 | 16.1 | 9.2 | 25.0 |  | 180.2 | 167.6 | 193.1 | 63.5 | 48.6 | 80.2 |
| 1986-1990 | 42.3 | 36.6 | 48.3 | 15.3 | 8.9 | 23.4 |  | - | - | - | - | - | - |  | 48.2 | 42.1 | 54.7 | 24.3 | 16.0 | 34.3 |  | 158.4 | 147.2 | 170.0 | 72.5 | 57.6 | 89.2 |
| 1991-1995 | 40.5 | 34.8 | 46.5 | 12.9 | 6.6 | 21.1 |  | - | - | - | - | - | - |  | 42.9 | 36.9 | 49.3 | 44.5 | 32.6 | 58.2 |  | 141.4 | 130.6 | 152.7 | 102.1 | 83.5 | 122.5 |
| **Lower white collar** |  |  |  |  |  |  |  | - | - | - | - | - | - |  |  |  |  |  |  |  |  |  |  |  |  |  |  |
| 1981-1985 | 81.3 | 74.2 | 88.6 | 14.7 | 11.6 | 18.1 |  | - | - | - | - | - | - |  | 43.1 | 38.0 | 48.5 | 26.5 | 22.3 | 31.1 |  | 226.9 | 215.1 | 239.1 | 78.6 | 71.2 | 86.2 |
| 1986-1990 | 72.8 | 66.2 | 79.7 | 21.3 | 17.7 | 25.1 |  | - | - | - | - | - | - |  | 51.0 | 45.4 | 56.8 | 39.3 | 34.4 | 44.6 |  | 208.7 | 197.4 | 220.3 | 104.2 | 96.0 | 112.6 |
| 1991-1995 | 57.6 | 51.4 | 64.1 | 20.3 | 16.7 | 24.3 |  | - | - | - | - | - | - |  | 55.9 | 49.8 | 62.4 | 44.3 | 38.6 | 50.4 |  | 197.5 | 185.9 | 209.4 | 119.5 | 110.1 | 129.2 |
| **Upper blue collar** |  |  |  |  |  |  |  | - | - | - | - | - | - |  |  |  |  |  |  |  |  |  |  |  |  |  |  |
| 1981-1985 | 106.6 | 99.1 | 114.4 | 24.8 | 19.7 | 30.6 |  | - | - | - | - | - | - |  | 54.2 | 48.8 | 59.8 | 37.5 | 31.0 | 44.7 |  | 285.3 | 273.0 | 298.0 | 119.5 | 107.8 | 131.9 |
| 1986-1990 | 86.9 | 80.2 | 93.9 | 24.2 | 19.2 | 29.9 |  | - | - | - | - | - | - |  | 56.8 | 51.4 | 62.6 | 54.3 | 46.4 | 62.8 |  | 249.1 | 237.6 | 261.0 | 149.9 | 136.8 | 163.5 |
| 1991-1995 | 74.1 | 67.6 | 81.0 | 33.1 | 26.7 | 40.2 |  | - | - | - | - | - | - |  | 57.3 | 51.4 | 63.4 | 61.1 | 52.0 | 70.9 |  | 230.4 | 218.7 | 242.5 | 165.4 | 150.6 | 180.9 |
| **Lower blue collar** |  |  |  |  |  |  |  | - | - | - | - | - | - |  |  |  |  |  |  |  |  |  |  |  |  |  |  |
| 1981-1985 | 105.6 | 91.8 | 120.3 | 22.4 | 17.3 | 28.1 |  | - | - | - | - | - | - |  | 38.1 | 30.0 | 47.2 | 30.1 | 24.0 | 36.8 |  | 253.0 | 231.3 | 275.5 | 103.8 | 92.4 | 115.8 |
| 1986-1990 | 94.8 | 80.8 | 109.8 | 20.6 | 15.6 | 26.2 |  | - | - | - | - | - | - |  | 69.7 | 57.6 | 82.9 | 50.9 | 42.5 | 60.0 |  | 284.2 | 259.4 | 310.1 | 135.8 | 122.2 | 150.2 |
| 1991-1995 | 96.6 | 80.2 | 114.6 | 30.0 | 22.9 | 37.9 |  | - | - | - | - | - | - |  | 61.4 | 47.9 | 76.6 | 54.3 | 44.1 | 65.5 |  | 295.6 | 266.0 | 326.8 | 151.9 | 134.8 | 170.0 |
| **Farmers/Forestry/Fishing** |  |  |  |  |  |  |  | - | - | - | - | - | - |  |  |  |  |  |  |  |  |  |  |  |  |  |  |
| 1981-1985 | 46.1 | 39.7 | 53.0 | 6.1 | 2.8 | 10.8 |  | - | - | - | - | - | - |  | 15.8 | 12.1 | 19.9 | 12.7 | 7.5 | 19.2 |  | 119.8 | 109.3 | 130.8 | 40.2 | 30.4 | 51.3 |
| 1986-1990 | 44.0 | 37.5 | 51.1 | 7.6 | 3.8 | 12.7 |  | - | - | - | - | - | - |  | 26.4 | 21.3 | 32.1 | 17.4 | 11.2 | 25.0 |  | 119.6 | 108.5 | 131.3 | 46.5 | 36.0 | 58.3 |
| 1991-1995 | 35.0 | 28.3 | 42.5 | 12.3 | 5.8 | 21.1 |  | - | - | - | - | - | - |  | 26.7 | 20.5 | 33.7 | 19.1 | 12.1 | 27.8 |  | 104.4 | 91.9 | 117.8 | 52.8 | 39.4 | 68.1 |
|  |  |  |  |  |  |  |  |  |  |  |  |  |  |  |  |  |  |  |  |  |  |  |  |  |  |  |  |
